# Airway Management during Thyroidectomy for a Giant Goitre due to McCune-Albright Syndrome

**DOI:** 10.1155/2018/4219187

**Published:** 2018-05-06

**Authors:** Hiroyuki Nakao

**Affiliations:** Department of Emergency and Critical Care Medicine, Hyogo College of Medicine, Hyōgo Prefecture, Japan

## Abstract

There have been no case reports to date describing the technical aspects of tracheal intubation in a patient with a goitre associated with McCune-Albright syndrome (MAS), even though goitre is frequently observed in this condition. I describe a case of resection of a giant goitre in a patient with MAS, with difficult airway management. Preoperative investigation showed that the trachea was shifted to the right by the goitre, with the narrowest part of the tracheal lumen 4 mm in diameter. There was dome-shaped protuberance of the posterior pharyngeal wall into the airway. The patient had an S-shaped total spine, a short neck, and a relatively large jaw, which interfered with airway visualisation during intubation. Anaesthesia was induced with light sedation and supplemental oxygen. Endotracheal intubation was successfully performed using a fiberoptic laryngoscope and a flexible, spiral-wound, obtuse-tipped tracheal tube.

## 1. Introduction

General anaesthesia in high-risk patients with anatomical anomalies of airway needs careful airway management, especially in cases undergoing prolonged neck surgery. I describe anaesthesia in a case of McCune-Albright syndrome (MAS) undergoing thyroidectomy. MAS is thought to be due to a point mutation of the GNAS1 gene on chromosome 20q13.2 and is characterised by polyostotic fibrous dysplasia, autonomous endocrine hyperfunction, and abnormal skin pigmentation (café-au-lait spots) [1, 2]. Patients may demonstrate precocious puberty, pathological fractures, bone malformations, pituitary gigantism, Cushing syndrome, or hypophosphatemia. In some cases, hyperplasia of the thyroid gland may compress the trachea [1–3]. However, there are no previous reports of general anaesthesia management in a MAS patient with a giant goitre.

I report a MAS patient with difficult airway management due to giant goitre, craniofacial deformities, extreme spinal deformities, and tracheal displacement and compression.

## 2. Case Presentation

A 33-year-old male who was diagnosed with MAS suffered from dyspnoea at bedtime for several months and was diagnosed with giant goitre at another clinic.

**Figure 1:**
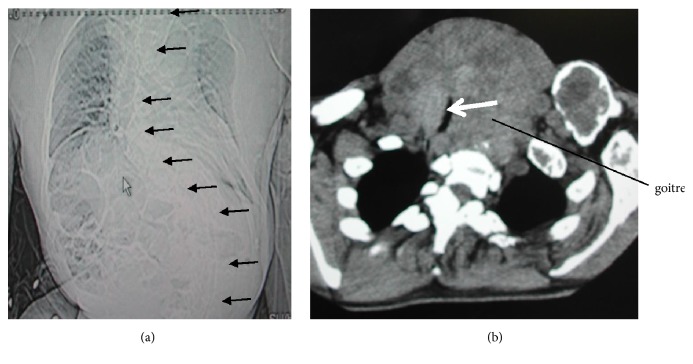
Preoperative CT scan. (a) Lateral curvature of the spine. (b) Narrowest portion of the trachea was 4 mm in diameter in lying down.

Physical examination showed a height of 145 cm and weight of 50 kg. He had skeletal abnormalities including craniofacial deformities, short neck, enlarged and greatly deformed thorax, scoliosis, and kyphosis (Figure 1(a)). He was unable to walk without help and could not be with face-up position for an airway obstruction. Patient's ASA physical status was class 3. His range of cervical spine movement was flexion 4° and extension 84° with measurement in his surface of a body. Palpation revealed a huge thyroid gland, but more specific thyroid examination was not possible due to his enlarged thorax and short neck.

**Figure 2:**
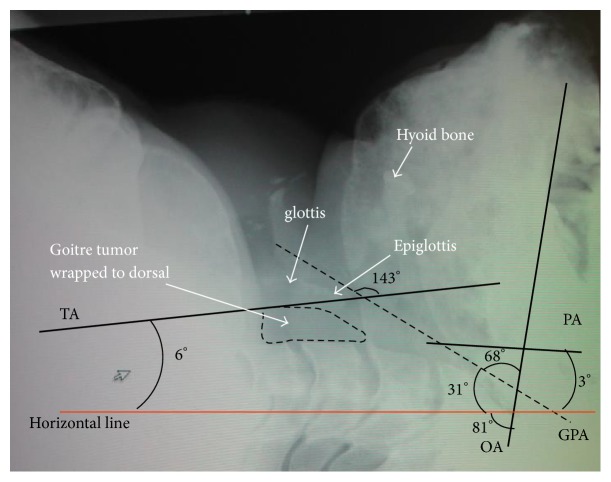
Preoperative lateral neck X-ray with the head resting on the bed in a neutral position. X-ray shows short neck with limited extension. The glottis is elevated by the dorsally located goitre. OA: oral axis, PA: pharyngeal axis, TA: tracheal axis, and GPA: glottis- pharyngeal axis.

Complete blood count, biochemistry, and arterial blood gas tests were normal. Thyroid hormone levels were mildly elevated (fT4 1.62 (0.7–1.48) ng/dl, fT3 3.50 (1.71–3.71) pg/ml, TSH 4.03 (0.35–494) *μ*g/dl, and T4 12.5 (4.87–11.7) *μ*g/dl). Lateral neck X-ray showed a dome-shaped protuberance of the posterior pharyngeal wall. The oral, pharyngeal, and tracheal axes were measured (Figure 2). Preoperative computed tomography (CT) scan showed that the trachea was shifted to the right and compressed by a giant goitre, with a lumen of 4 mm × 14 mm at its narrowest point in the subglottic region. The narrow portion extended over a length of about 20 mm (4 slices at 5 mm width) (Figure 1(b)). The cross-sectional oval area was 2 × 7 × 3.14 = 43.96 mm^2^. The cross-sectional orbicular area was 4.35 × 4.35 × 3.14 = 59.42 mm^2^. The goitre had a benign appearance on ultrasonography and I assumed that goiter was softer than malignant tumor. I could anticipate that an endotracheal tube could enlarge a narrow soft tumor. In laryngeal fiberscope of the preoperative examination, I recognized his elevated lesion in the posterior pharyngeal wall and his vocal cord shifted to right side. Total thyroidectomy was scheduled and was expected to take approximately 8 hours.

Intraoperative monitoring included ECG, noninvasive and invasive blood pressure, end-tidal CO_2_, bispectral index (BIS), and pulse oximetry. Because this case was predicted to have a difficult intubation, general anaesthesia was cautiously induced with oxygen and fentanyl (0.1 mg of the total dose), and intubation was performed with the patient semiawake. No muscle relaxants were used, and spontaneous ventilation was preserved. The patient was given a sufficient preoperative explanation of semiawake intubation to eliminate anxiety. After the operation, I confirmed that he did not remember the intubation even though the BIS was maintained within the 95–98 range.

Our plans for intubation were (1) attempt intubation with conventional laryngoscopy, (2) attempt intubation with flexible fiberscope laryngoscopy and a variety of endotracheal tubes, and (3) laryngeal mask airway (LMA) for ventilation if the operation was abandoned.

LMA is not used in thyroidectomy at the thyroid position that the neck is greatly extended increasing the space between the clavicles and the jaws for his giant goiter, or with risk of bilateral recurrent nerve paralysis on operating. The operation cannot be carried out if plans 1 and 2 did not succeed. Plan 3 was prepared for his safety based on ASA difficult ventilation guideline until he wakes up.

**Figure 3:**
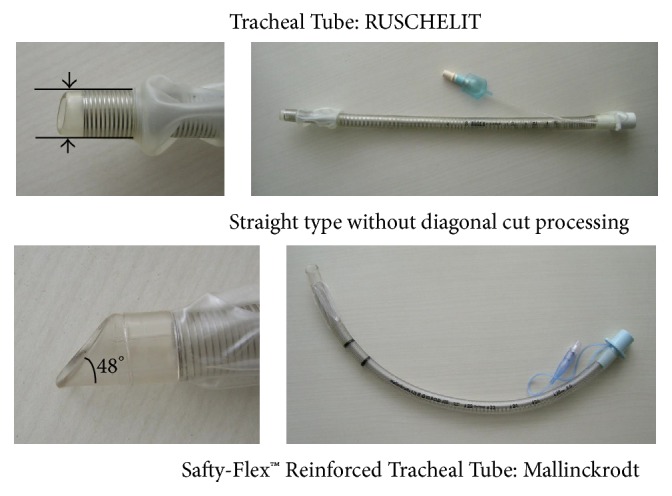
Straight type without diagonal tip. The Rüschelit tube has an obtuse-angled tip; the Safety-Flex Reinforced tube has a slanted tip.

**Figure 4:**
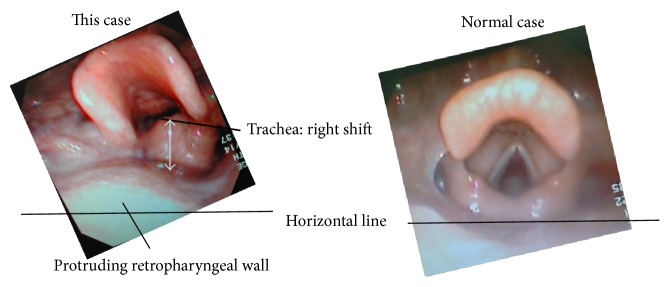
Preoperative laryngeal fibroscopy showing the trachea shifted to the right, and the posterior pharyngeal wall and glottis were pushed forward.

Oral intubation could not be accomplished using a conventional laryngoscope (Macintosh curved blade) as it was impossible to elevate the protruded epiglottis (Cormack and Lehane Grade 3). It was very difficult to identify intraoral structures by fiberscope. I chose a fiberscope for high torque capability (Olynpus laryngofiberscope ENF-V2: OD 3.2 mm, Up/Down 130°/130°). I attempted nasotracheal fiberoptic intubation with a standard endotracheal tube (Lo-counter™ Muphy Tracheal Tube Mallinckrodt, 5.5 mm internal diameter (ID)) and a spiral-wound tube (Safety-Flex™ Reinforced Tracheal Tube Mallinckrodt, 5.5 mm ID) (Figure 3). Both tubes have angled tips. Even though I were able to introduce the fiberscope into the trachea, I were unable to perform tracheal intubation as I could not pass the tip of the endotracheal tube beyond the arytenoids (Figure 4).

**Figure 5:**
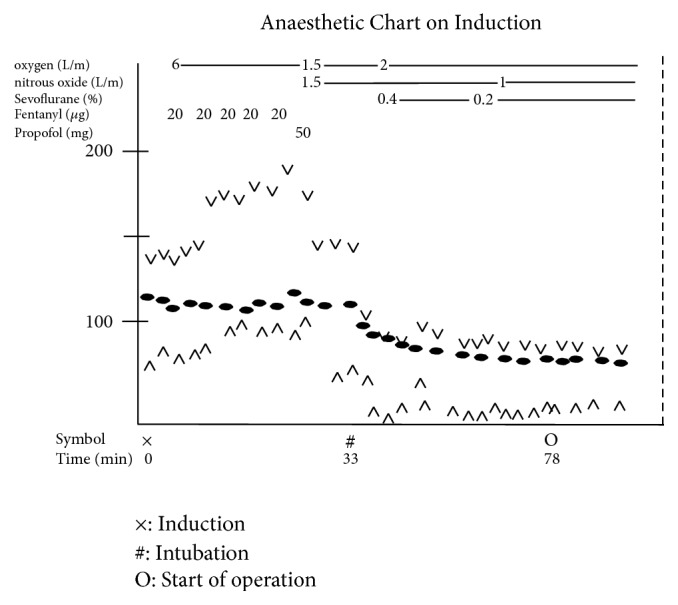
Anaesthetic chart of patient undergoing thyroidectomy. ×: induction, #: intubation, and O: start of operation.

**Figure 6:**
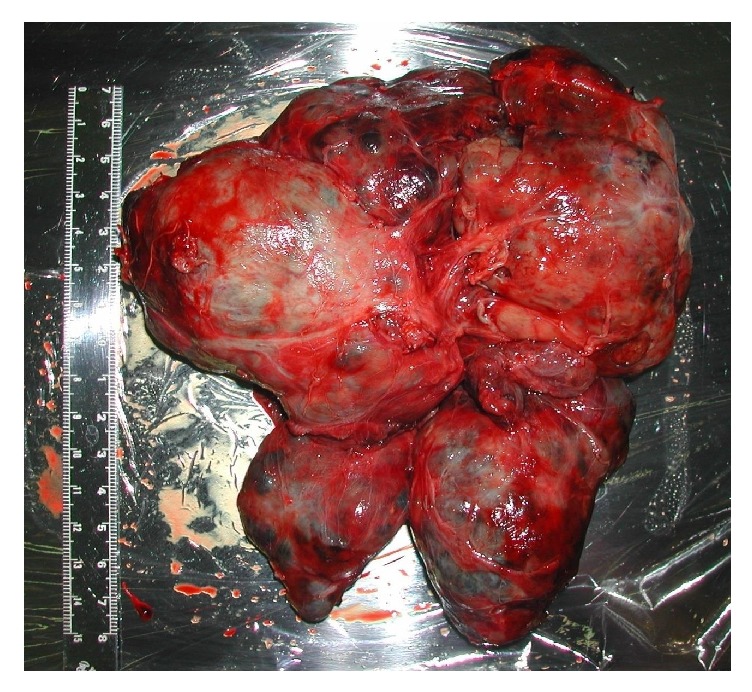
The extirpated tumour (515 g, 15 cm × 13 cm).

I proceeded to orotracheal intubation with fiberoptic laryngoscopy and a spiral-wound tube (Rüschelit Tracheal Tube, 5.5 mm ID) with an obtuse-angled tip (Figure 3). This method allowed us to successfully perform intubation without being blocked by the arytenoids. The slight rightwards shifts of the trachea by the goitre did not increase the difficulty of intubation using the fiberoptic laryngoscope. The tumour was soft and permitted easy placement of the tracheal tube. The total anaesthetic time was 8 hours 32 minutes, with 33 minutes' tracheal intubation from induction of anaesthesia and 6-hour 49-minute operation (Figure 5). I did not extubate the patient immediately after the operation because of the risk of postoperative oedema of the glottis and neck. Examination by fibroscopy showed intact recurrent laryngeal nerve function. Extubation the following day was uneventful. The extirpated tumour weighed 515 g and measured 15 cm × 13 cm (Figure 6).

## 3. Discussion

Mastorakos and colleagues reported that goitre is observed in 71.4% of MAS cases [1]. The combination of goitre and skeletal malformations in MAS requires special attention to securing the airway during general anaesthesia [4, 5]. However, there are no previous reports describing general anaesthesia in an MAS patient undergoing thyroidectomy for a giant goitre. Bouaggad and colleagues reported that difficult tracheal intubation was likely in thyroid surgery cases with thyroid cancer, tracheal compression, or dyspnea [6]. This case demonstrates that a goitre which extends dorsally also causes difficult tracheal intubation because of elevation of the glottis. Careful preoperative evaluation by CT scan is therefore important in cases of giant goitre.

Short neck, obesity, round back, limited neck extension, enlarged thorax which encroaches on the face, abnormal facial structure, tracheal deviation, and tracheal compression may all contribute to technical difficulties with intubation in patients with MAS. All of these except for obesity were present in this case. Preoperative assessment included neck CT scan, neck X-rays, and fiberoptic laryngoscopy to check the entire length of the trachea. As a result, it was expected that technical intubation difficulties might be encountered and I undertook preoperative planning to secure the airway [7, 8]. The difficult airway algorithm of the American Society of Anesthesiologists (ASA) suggests the use of mask anaesthesia, laryngeal mask airway anaesthesia, local anaesthesia, or regional anaesthesia not requiring tracheal intubation in cases with technical intubation difficulties [9]. However, in a patient with a giant goitre, tracheal intubation is essential to secure the airway during a prolonged procedure with the neck retroflexed. In this case, the remarkably large size of the goitre would also interfere with the ability to perform prompt cricothyrotomy or tracheostomy.

When severe malformation of the face is present as in the present case, the use of a muscle relaxant may increase the difficulty of airway management due to relaxation of the muscles in the oral cavity [10, 11]. I also hoped that spontaneous respiration might help to localize the vocal cords due to air currents moving the saliva.

And novel video assisted device can observe the epiglottis and is useful for an expected intubation difficulty [12, 13]. These devices may fast observe his epiglottis in this case too. However, the trachea tube must get over the tumor around his glottides because a tumor piled up the glottides to a ventral from the dorsal. Therefore, as for Reinforce Tracheal Spiral Tube that the tip was cut keenly, the tube cut surface touched the trachea ventral wall, and the tube could no longer advance.

It is important to consider the oral, pharyngeal, and tracheal axes during endotracheal intubation. Bannister and Macbeth reported that alignment of these three axes is important for successful intubation [14]. In this case, the goitre pushed the glottis ventrally, and I had to consider four axes during intubation: the oral axis which was 81° to the horizontal on X-ray at maximum retroflexion, the pharyngeal axis (3°), the tracheal (6°), and the glottic-pharyngeal axis (31°) (Figure 1). The endotracheal tube therefore had to be passed around the protrusion caused by the tumour.

Horton and colleagues reported the “ideal angles” for upper cervical flexion and lower cervical extension as 15° and 35°, respectively, which they determined by reviewing various values from previous research [15]. The upper cervical flexion angle of 4° in this case indicated restricted mouth opening, and the lower cervical extension angle of 84° indicated restricted neck extension.

I attempted to intubate with the patient's head elevated on a pad and his neck extended but was unable to align the axes due to his large jaw, short and inflexible neck, abnormally shaped thorax, and kyphosis. The fiberscope had to follow an S-shaped path with a 68° angle followed by a 143° angle, and the endotracheal tube then followed the path of the fiberscope. Successful intubation was helped by the pliability of the endotracheal tube and the shape of the tip which eventually enabled it to pass through the glottis.

Some researchers have reported on a variety of tracheal tubes with their tips designed especially for fiberoptic intubation of difficult airways [16–19]. Among them, the Rüschelit tracheal tube is flexible enough to follow the path of a laryngeal fiberscope and is able to get past protrusion in the subglottis because of the obtuse-angled tip (Figure 4) [20].

In general, the difficulty of tracheal intubation in cases with thyroid disease is affected by compression of the trachea and the position and hardness of the tumour [21, 22]. In this case, the position of the tumour posterior to the trachea increased the difficulty of intubation. However, the soft texture of the tumour made it easy to pass the tube through the narrowed part of the trachea.

In the present case with MAS complicated by giant goitre and skeletal malformations, successful intubation was achieved with detailed assessment, careful induction, and appropriate selection of endotracheal tube. The most useful tool in achieving this difficult intubation case was the fiberoptic laryngoscope which helped to identify the glottis.
